# Implementation, barriers, solving strategies and future perspectives of reimbursed community pharmacy services - a nationwide survey for community pharmacies in Germany

**DOI:** 10.1186/s12913-024-11745-y

**Published:** 2024-11-25

**Authors:** Ann-Christin Kroenert, Thilo Bertsche

**Affiliations:** 1 Department of Clinical Pharmacy, Institute of Pharmacy, Medical Faculty, Leipzig University, Leipzig, Germany; 2 Drug Safety Center, Leipzig University, Leipzig, Germany; 3 Clinical Pharmacy, Institute of Pharmacy, Medical Faculty, and Drug Safety Center, Leipzig University and Leipzig University Hospital, Brüderstraße 32, Leipzig, 04103 Germany

**Keywords:** Community pharmacies, Community Pharmacy services, Pharmacists, Community pharmacists, Survey, Germany

## Abstract

**Background:**

Since June 2022, the legal framework has been created for German community pharmacies to offer their patients five reimbursed community pharmacy services that go beyond the current operating regulations. However, little is known about barriers that hinder their implementation. We therefore aimed to investigate the implementation of reimbursed community pharmacy services (i), barriers to the implementation (ii), solving strategies to overcome the barriers (iii), and future perspectives (iv). The objective of this study is to find out how the implementation of community pharmacy services can be facilitated for community pharmacies so that more services can be offered.

**Methods:**

In July 2023, we created an online survey and sent it to pharmacists in community pharmacies, including those who offered reimbursed community pharmacy services and those who did not.

**Results:**

Overall, 218 pharmacists from 218 different community pharmacies participated. (i) Of those, 176 (81%) already offered at least one reimbursed community pharmacy service. (ii) For hypertension service, 33% of the offering pharmacists reported barriers in “Communicating with patients,” and 41% reported “Too little patient demand.” For polymedication service, 53% of the offering pharmacists indicated “Barriers in communication with physicians,” and 44% mentioned “Fear of competing with physicians.” (iii) The most frequently reported solving strategies of pharmacists in offering pharmacies were taking advanced training (median of all five services 42%) and developing standardized procedures (median of all five services 34%). In contrast, pharmacists in non-offering pharmacies had not developed any solving strategies (median of all five services 40%). (iv) 64% of the pharmacists in non-offering pharmacies could imagine being able to offer reimbursed community pharmacy services in the future.

**Conclusions:**

Many German pharmacies already offer reimbursed community pharmacy services. However, there are still barriers to widespread implementation. Therefore, customized support regarding the needs of the pharmacies should be provided since most pharmacists who do not yet offer these services today can imagine offering them in the future.

**Supplementary Information:**

The online version contains supplementary material available at 10.1186/s12913-024-11745-y.

## Background

The professional activity of pharmacists in community pharmacies is changing. In addition to dispensing drugs and extemporaneous drug preparation, patient counselling is becoming increasingly relevant in this setting. Understanding of patient- and drug-related influences on pharmacodynamic and pharmacokinetic parameters (including pharmacogenetics) has deepened through comprehensive research. If this sophisticated knowledge is not considered when making therapeutic decisions, prescribing errors are likely, for example if drug dosing is not tailored to the individual organ functions under routine conditions [1]. In addition, innovative drug formulations are more complicated to administer which is why the risk of drug handling and administration errors increases [2]. As part of evidence-based medicine, effectiveness under real world conditions also depends largely on ensuring sufficient patient adherence [3]. However, highly effective therapies are also associated with risks, such as adverse drug reactions or drug-drug interactions, that can be avoided by involving pharmaceutical expertise [4]. Pharmacists in a community pharmacy contribute to this with their specialist expertise and promote drug therapy safety together with the general practitioner in primary care [5]. The low-threshold contact at the pharmacy means that drug related problems can be recognized quickly before they become clinically relevant and require a consultation with the general practitioner [6]. In this context, preventive approaches aimed at maintaining health or avoiding (further) events in the case of corresponding pre-existing conditions (secondary and tertiary prevention) play a special role [7].

Studies have shown that pharmacists in community pharmacies make an important contribution to medication safety, especially in the case of chronic illnesses such as high blood pressure or asthma [8, 9]. Patients-reported outcomes such as “Quality of Life” could be improved in this way [10]. In Germany, as in the European Union, advice and information on medicines in the community pharmacy are based on legal requirements [11]. Comprehensive pharmaceutical counselling, which has achieved particularly impressive results in model projects with a lower risk of death [12], shows that the effort involved in such community pharmacy services is worthwhile in terms of patient safety.

From a health economic perspective, it makes sense to involve pharmacists more in drug therapy (also resulting in deprescribing) rather than merely selling products [13]. In this way, the healthcare system can benefit from pharmaceutical expertise, and resources can be used more specifically for patients’ benefit.

As a consequence of those considerations, reimbursed community pharmacy services were introduced in Germany in June 2022 by the Law on Strengthening Local Community Pharmacies. Statutory and privately insured patients of German community pharmacies are legally entitled to five different reimbursed community pharmacy services that go beyond the previous operating regulations. The first community pharmacy service is “Blood pressure control in hypertension” (**Hypertension)**. This service can be provided to patients taking at least one antihypertensive drug at least for two weeks by all pharmaceutical dispensing staff. It is remunerated to the pharmacy with 11.20 EUR + VAT. The second one is “Assuring proper inhalation techniques for patients receiving a new device or a device change” (**Inhalation)**. This service can be provided to patients from the age of six who receive a new device or a device change by all pharmaceutical dispensing staff except from those still in training. It is remunerated to the pharmacy with 20 EUR + VAT. The third service is “Medication review for patients with polymedication” (**Polymedication)**. This service can be provided to all patients taking at least five different prescribed drugs by pharmacists who finished an advanced training. It is remunerated to the pharmacy with 90 EUR + VAT. The last two services, “Medication review with follow-up for patients taking immunosuppressants post-transplantation” **(Transplantation)** and “Medication review with follow-up for patients taking oral anticancer drugs” **(Anticancer drugs)**, contain a Medication review with special attention to the specific medication of anticancer drugs or immunosuppressants. They can be provided to all patients taking immunosuppressants post-transplantation or taking oral anticancer drugs by pharmacists who finished an advanced training. These services are remunerated to the pharmacy with 90 EUR + VAT. A follow-up consultation after two to six month is remunerated to the pharmacy with 17.55 EUR + VAT.

All services can be provided once in 12 month or when there is a substantial change in prescribed medication [14]. To improve the readability, we use the bold short terms when discussing the five community pharmacy services.

For all those five services the pharmacist has to fill in a documentation form. Additionally the patient has to fill in a data protection declaration, has to sign that he received the service and, if needed, the patient has to fill in a form that releases the physician from his duty of confidentiality.

The reimbursement is regulated via a fund managed by the Night and Emergency Service Fund. This fund is paid by the statutory and privately insurance companies, in which patients are mandatory members in Germany. Its amount is 150 million EUR per year and it is additional to the standard reimbursement system for drug dispensary [14]. At the time of this study the invoicing of the community pharmacy services had to be done in paper form.

The content of the services was negotiated by the National Association of statutory health insurance funds and the German Pharmacists’ Association, who is responsible for representing the economic and commercial interests of the German community pharmacies [15].

Diverse community pharmacy services have been provided in the recent years in several countries worldwide with different remuneration models. One model is the so called “fee-for-service”, as it is used in Germany. Here a pharmacy is paid a fixed fee for every community pharmacy service provided. Another model is the so called “Capitation”. This service is used for example in the US. The pharmacy is paid for every patient registered with the pharmacy, no matter which services and how many services are provided. The third model is the “blended funding model” which is a combination of the two models explained before. It is for example used in Australia.

In many countries, the implementation of community pharmacy services is evaluated according to the models used in the country concerned [16]. Therefore, the question arises as to the extent to which reimbursed community pharmacy services are actually implemented in community pharmacies in Germany. Barriers should be identified to improve the implementation rate, and solutions should be proposed to clearly define community pharmacy services’ future perspectives. Therefore, we aimed to investigate the status quo of reimbursed community pharmacy services via an online survey offered to pharmacists working in community pharmacies in Germany. We asked about the implementation of reimbursed community pharmacy services (i), barriers to the implementation (ii), solving strategies to overcome the barriers (iii), and future perspectives concerning reimbursed community pharmacy services (iv). The aim of our study was to gain a better understanding of why some pharmacies successfully offer remunerated services, but also why other pharmacies have not yet done so. We consider both findings to be important in order to be able to optimize the framework conditions for pharmacies in the future, but also to provide pharmacies with their own opportunities to offer the services more frequently.

## Methods

### Ethics approval

The study protocol was approved by the ethics committee of the Medical Faculty of the University of Leipzig in May 2023 (#128/23-ek). The participating community pharmacies were informed about data safety and anonymous data collection prior to the start of the online survey. No data concerning the pharmacies, such as IP address, location or participation time, have been saved. The survey could have been stopped at any time, but data deletion was not possible after the survey had been finished. By starting the survey, participants gave their informed consent to the anonymous data collection.

### Study design

We developed an online survey as a prospective status quo analysis about the reimbursed community pharmacy services in Germany. We asked about the implementation of reimbursed community pharmacy services (i), barriers to implementing them (ii), solving strategies to overcome the barriers (iii), and future perspectives concerning reimbursed community pharmacy services (iv). The study was designed to question pharmacists in pharmacies that were already offering the services as well as those who had not yet offered them. From each pharmacy, only one pharmacist should participate.

### Online survey

The questions of the online survey were created by an expert panel consisting of internal (from Leipzig University) and external experts on the subject. Their understandability and relevance were pretested in a group of internal pharmacists independent of the main survey. In this context, questions and formulations of questions were modified for better understandability. Subsequently, we performed a pilot test in pharmacies experienced with reimbursed community pharmacy services (namely co-operating community pharmacies in the area of Leipzig). Again, the appropriateness and usefulness of the questions were checked. Data generated in the pilot test was not included in the final results of the study.

The Survey was separated into the following four parts:

- **i. Implementation**: In the first part of the survey, participating pharmacists were asked whether reimbursed community pharmacy services were currently offered in their pharmacies. Pharmacists who checked “Yes” were asked which reimbursed community pharmacy services they offered. Pharmacists who checked “No” were asked if any services similar to the reimbursed community pharmacy services were provided in their pharmacy but not billed.
- **ii. Barriers**: In the second part of the survey, we asked about barriers that came up in the implementation process or that still impeded the implementation of reimbursed community pharmacy services. These barrier questions were asked of all pharmacists, whether they were offering services or not. Each pharmacist was asked about the barriers to the five different reimbursed community pharmacy services. We identified 17 barriers that may have come up in implementing the services and may have impeded the implementation so far. Additional free text options were offered. Multiple categories could be chosen. In the following, we will only display the most important categories; the remaining ones can be seen in the supplementals.
- **iii. Solving strategies**: In the third part, we asked which solving strategies the pharmacists developed to facilitate the implementation of the reimbursed community pharmacy services. Several answers were suggested with additional free text options; again, multiple categories were possible.
- **iv. Future perspectives**: Finally, we asked how the offer of reimbursed community pharmacy services might develop in the future. Here, only one answer could be selected. Additionally, we asked what further support the pharmacists would wish for in the future to increase the number of reimbursed community pharmacy services offered. For this, multiple categories could be chosen, and additional free text options were offered.

In all parts of the survey, the answers have been divided into pharmacists working in pharmacies that already offer the specific reimbursed community pharmacy service and those who were not (yet) offering it.

The survey has been designed for this research. The structure of the survey can be seen in supplemental 1.

### Participants and setting

The online survey was offered to all German chambers of pharmacists for sending it to their member pharmacists. In Germany, all pharmacists are compulsory members of one of the 17 chambers of pharmacists [15].

Data collection was performed from July 2023 until December 2023. Reminder E-Mails were sent by the German chambers of pharmacists, who agreed to send the survey link to their members, in October and November 2023. In addition, the link to the online questionnaire was published in the central organ of the Federal Union of German Associations of Pharmacists in August 2023 to reach all community pharmacies that could not be reached via their chambers of pharmacists. Therefore all 17,571 German community pharmacies (number of community pharmacies by the End of 2023) [17] were invited to participate.

In Order to avoid influence within the team and to be able to evaluate as many different pharmacies as possible, only one person per pharmacy was allowed to participate. The participation was independent of whether they already offered the services or not.

### Statistics and data evaluation

The answers were exported from LimeSurvey and entered into Microsoft Office Excel 2019 MSO (16.0.10409.20028) 64 Bit (Microsoft Corporation, Redmond, Washington, USA) and IBM SPSS Statistics Version 29.0 (IBM Corporation, Armonk, New York, USA) for data analysis. The absolute (N) and relative (%) frequencies of the nominal and ordinal data were determined. Missing data were described as ‘no answer’. The order of the data in the graphics corresponds to the order in the survey.

## Results

### Participants

In total, 218 pharmacists, each working in 218 different German community pharmacies, completed the online survey. The participating pharmacies came from all German chambers of pharmacists (allocation see Table 1).

**Table 1:** Participating pharmacists, grouped by federal state and whether or not their pharmacy offers at least one of the defined reimbursed community pharmacy services

Federal state	Number of participating pharmacists from offering pharmacies	Number of participating pharmacists from NON-offering pharmacies	Total number
Baden-Wuerttemberg	14	7	21
Bavaria	0	1	1
Berlin	0	1	1
Bremen	3	0	3
Brandenburg	11	2	13
Hamburg	2	1	3
Hesse	5	0	5
Mecklenburg-Western Pomerania	28	7	35
Lower Saxony	2	0	2
North Rhine	12	1	13
Westphalia-Lippe	2	0	2
Rhineland-Palatinate	1	0	1
Saarland	1	0	1
Saxony	52	7	59
Saxony-Anhalt	34	9	43
Schleswig-Holstein	1	0	1
Thuringia	6	2	8
No specification	2	4	6

### Implementation (i)

Of the 218 participants, 176 (81%) already offered at least one of the defined reimbursed community pharmacy services in their pharmacy. 160 (94%) of these 176 pharmacists offered services for hypertension, 157 (92%) for inhalation, and 152 (89%) for polymedication. 23 (13%) offered services for transplantation, and 31 (18%) for anticancer drugs. 20 pharmacists (11% of the offering pharmacists) stated that their pharmacy already offered all five service.

28 (67%) of the 42 pharmacists working in non-offering pharmacies already offered similar services concerning blood pressure measurement, and 18 (43%) performed a detailed guide for inhalation techniques using demo inhalers. A drug interaction check was performed by 23 (55%) of the non-offering pharmacies, while 5 (12%) offered full medication analyses. 8 (19%) of the non-offering pharmacies reported not offering any similar services so far.

### Barriers (ii)

**Fig. 1:**
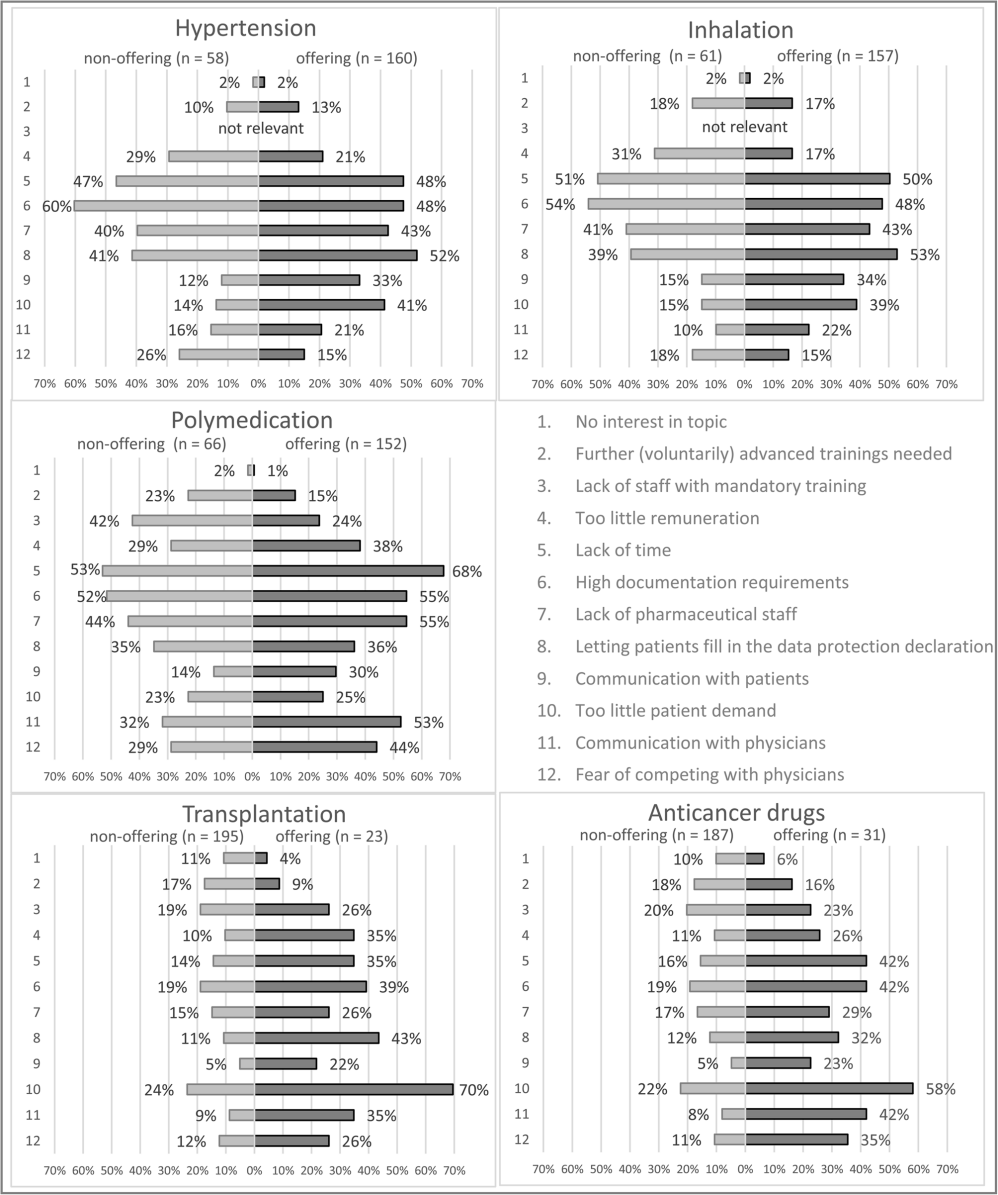
Barriers for the implementation of the reimbursed community pharmacy services comparing pharmacists working in offering and those working in non-offering community pharmacies - Multiple categories could have been chosen. On the right-hand side, we present the responses from pharmacists, who have already offered the service in question. On the left-hand side, we present the responses from pharmacists who have not yet offered the service in question

As shown in Fig. 1, the most often mentioned barriers were a lack of time, a lack of pharmaceutical staff and the high documentation requirements. Pharmacists also considered it a hindrance that patients have to fill in a data protection declaration before the service can be provided.

Additionally, 66 (41%) pharmacists in offering pharmacies reported too little patient demand for hypertension service and 61 (39%) for inhalation service. 16 (70%) pharmacists in offering pharmacies and 46 (24%) in non-offering pharmacies reported this barrier concerning transplantation service, and 18 (58%) pharmacists in offering pharmacies and 42 (22%) in non-offering pharmacies reported this barrier concerning anticancer drugs service.

17 (29%) pharmacists in non-offering pharmacies considered the remuneration for the hypertension service to be too low, as did 19 (31%) for inhalation service. For polymedication, 80 (53%) pharmacists in offering pharmacies reported a barrier to communicating with the physician, and 67 (44%) reported fear of competing with the physician.

### Solving strategies (iii)

**Fig. 2:**
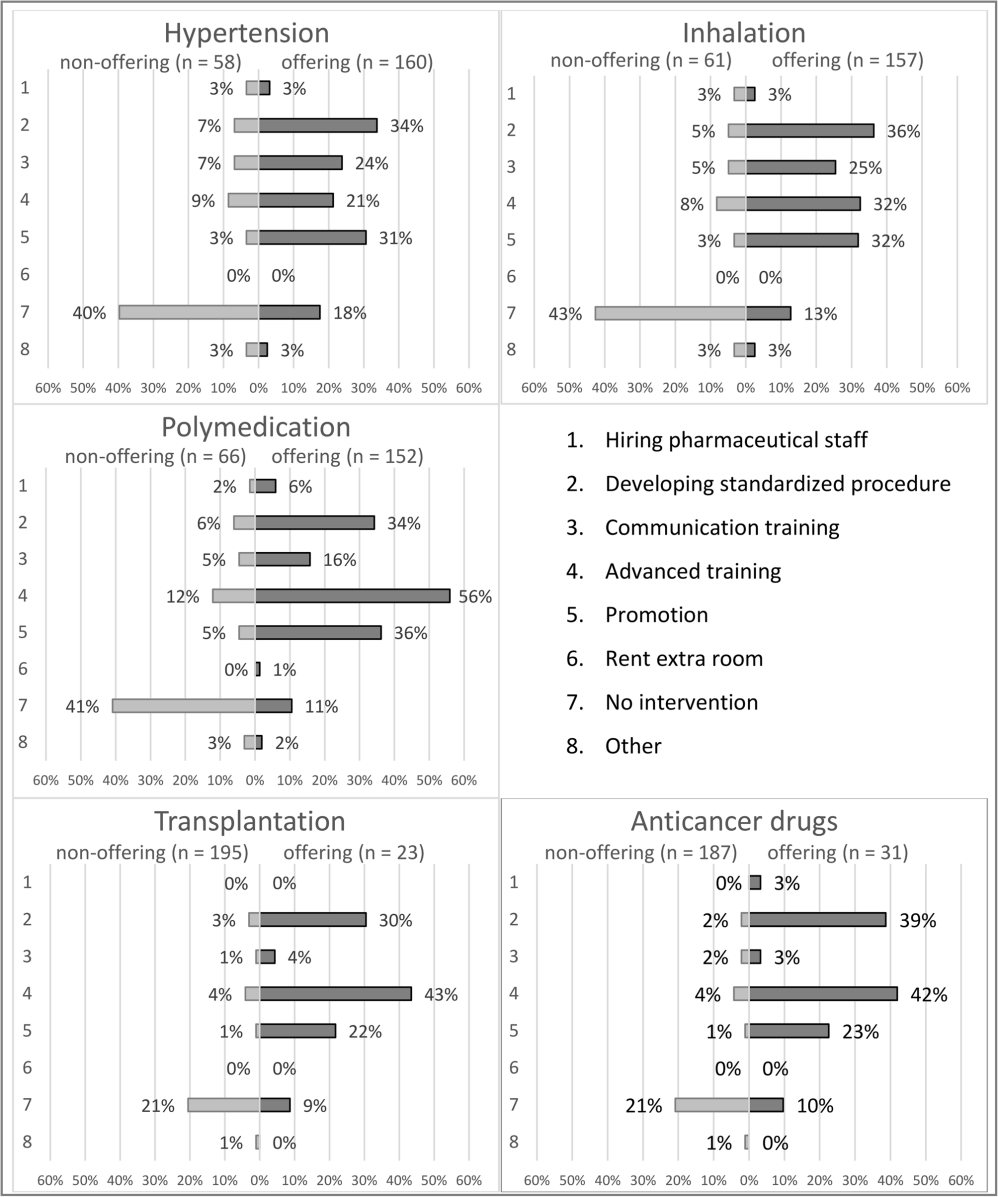
Solving strategies of the community pharmacies to overcome the barriers compared between pharmacists working in offering and non-offering community pharmacies - Multiple categories could have been chosen

As shown in Fig. 2, the most frequently given answer from pharmacists in non-offering pharmacies was that they had not tried any solving strategies to overcome the barriers to implementing the reimbursed community pharmacy services (median of all five services 40%).

Pharmacists in offering pharmacies have developed a standardized procedure for the community pharmacy services (median of all five services 34%). 49 (31%) have promoted the hypertension service and 50 (32%) the inhalation service. 85 (56%) pharmacists in offering pharmacies had advanced training for polymedication service, 10 (43%) for transplantation service and 13 (42%) for anticancer drugs service.

The fewest pharmacies had hired new staff (median of all five services for pharmacists in offering pharmacies 3%; median of all five services for pharmacists in non-offering pharmacies 2%), while 13 pharmacists (6% of all participating pharmacists) commented that they had difficulty finding pharmaceutical staff.

### Future perspectives (iv)

**Fig. 3:**
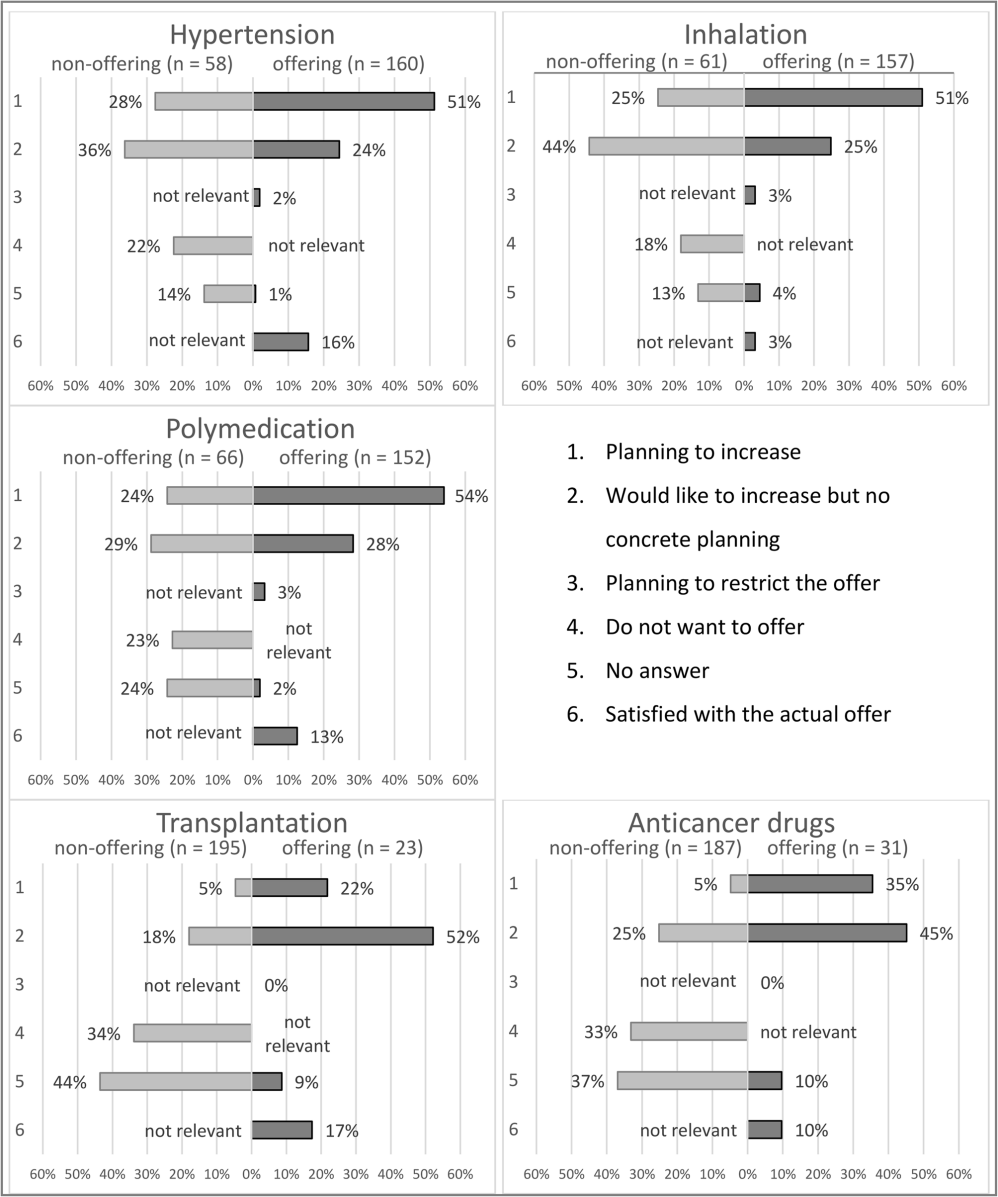
Plans for future development of the offer of reimbursed community pharmacy services compared between pharmacists working in offering and non-offering community pharmacies – Only one category could be chosen

As shown in Fig. 3, for the future, 82 (51%) pharmacists in offering pharmacies are planning to increase the number of services for hypertension, 80 (51%) for inhalation and 82 (54%) for polymedication. Very few want to reduce the number of reimbursed community pharmacy services offered (median of all five services 2%).

27 (64%) pharmacists in non-offering pharmacies would like to offer reimbursed community pharmacy services in the future, especially those concerning hypertension, inhalation, and polymedication. 66 (34%) pharmacists in non-offering pharmacies do not want to offer the service for transplantation, nor do 62 (33%) for anticancer drugs.

**Fig. 4:**
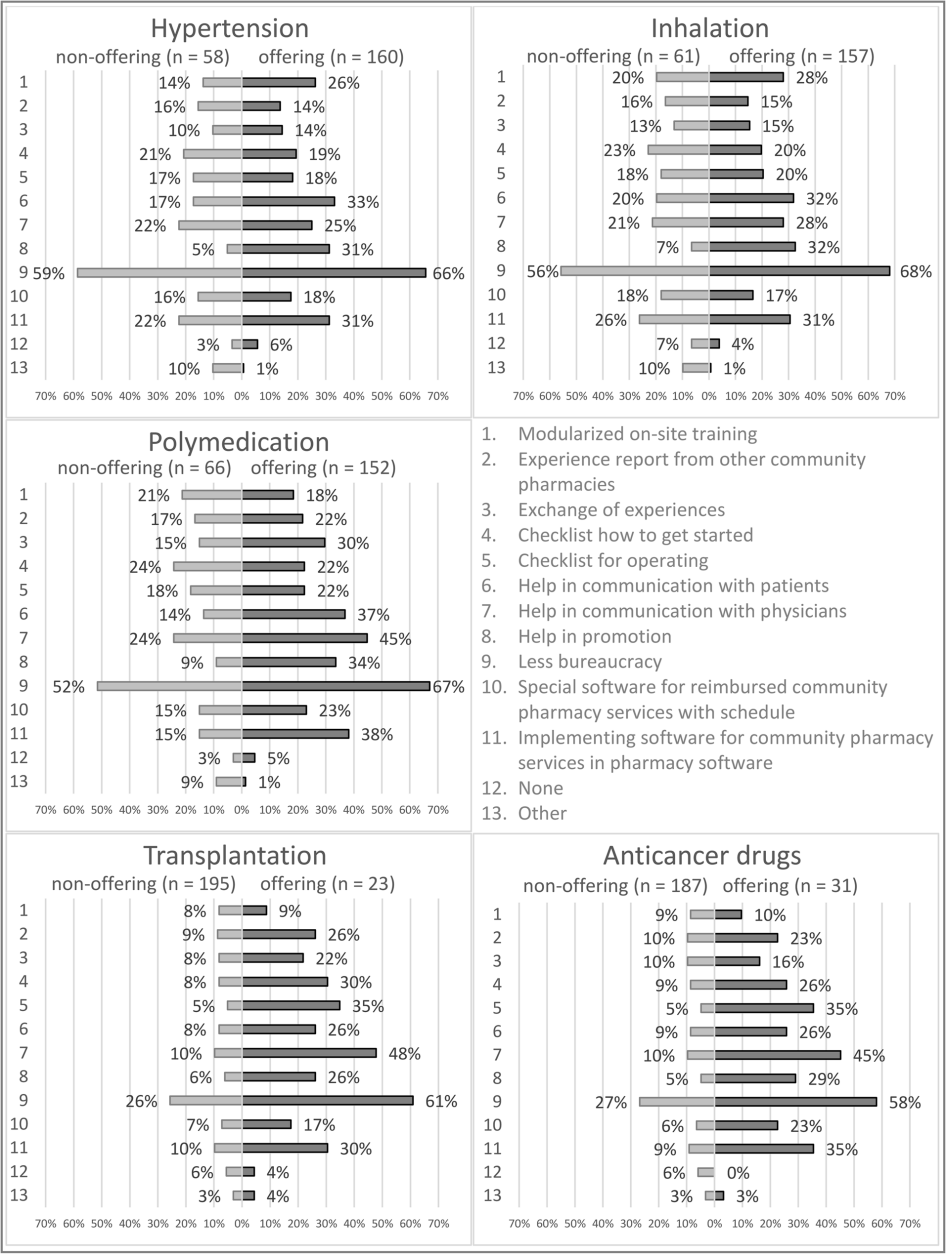
Wishes for support compared between pharmacists working in offering and non-offering community pharmacies - Multiple categories could be chosen

As shown in Fig. 4, most pharmacists, from offering (median of all five services 66%) and non-offering pharmacies (median of all five services 52%), ask for less bureaucracy.

53 (33%) pharmacists offering the hypertension service would like to receive support in communicating with patients. 50 (32%) pharmacists offering the inhalation service wish for the same support. 68 (45%) pharmacists in offering pharmacies wish for help in communicating with physicians concerning polymedication service, 11 (48%) about transplantation service and 14 (45%) regarding anticancer drugs service.

In the free text, the participating community pharmacists reported that they would offer nutritional advice and help diabetics with blood sugar measurement, pen handling, and insulin pump use if those pharmacy services were reimbursable. In addition, it was reported that dealing with the unavailability of medicines should be better paid for and that the payment to community pharmacies in general needs to be adjusted before more reimbursed community pharmacy services can be offered.

## Discussion

### General considerations

The newly reimbursed community pharmacy services have the potential for increasing patient safety [8, 9, 12]. This potential should now be used in practice, and the services should be offered to patients across the board. Therefore, in this project, we have performed a sophisticated analysis to characterize indicators why the implementation (i) in certain pharmacies has already been successful as well as to identify the barriers why these services have not yet been offered in some pharmacies (ii). The focus was also on solving strategies to overcome these barriers (iii). The future perspectives with regard to enhancing reimbursed community pharmacy services (iv) also formed a further focal point.

#### Implementation (i)

In this survey, 81% of the participating community pharmacists work in pharmacies that offer at least some reimbursed community pharmacy services, and 19% work in pharmacies that have yet to offer any such services.

There is a clear separation between three frequently offered services and two rarely offered services: Nine of ten offering pharmacies provide hypertension, inhalation, and polymedication services, whereas transplantation and anticancer drugs services are provided by fewer than one in four.

This is consistent with the trend of the billing data from the Night and Emergency Service Fund in the third quarter of 2022 [18], where the most often offered reimbursed community pharmacy service addressed inhalation, followed by hypertension and polymedication. Transplantation and anticancer drugs services barely appeared [18].

Irish data underline the high number of services concerning polymedication, hypertension, and inhalation, reporting a similar use of those services in 2016 [19].

#### Barriers (ii)

In all reimbursed community pharmacy services, the pharmacists face the barriers of high documentation effort, having the patients fill out the data protection declaration and a lack of time and pharmaceutical staff.

For hypertension and inhalation, pharmacists in offering pharmacies observe that patient demand is too low and communication with patients is a barrier. The communication barrier may amplify the low demand. Additionally, the low demand may arise because the reimbursed community pharmacy services can only be offered once a year if there are no relevant changes in medication.

Studies performed in the setting of community pharmacies have reported the benefit of community pharmacy services for example in patients with asthma bronchiale [8, 20]. Here, humanistic outcomes such as asthma specific quality of life and medication adherence improved through pharmaceutical care as well as asthma severity, peak expiratory flow, and patients’ inhalation technique. Other papers addressed the adherence to antihypertensives [21]. Therefore, positive results can be expected for a broad range of patients from a high implementation rate of inhalation and hypertension services. Additionally, this justifies the high (teaching and training) effort in the individual pharmacy for those services and makes further measures for increased implementation appear worthwhile from the perspective of the entire healthcare system.

Pharmacists of non-offering pharmacies describe too little remuneration as the main barrier to implementing hypertension and inhalation services. This is in line with former studies where 93% of participating community pharmacists reported that remuneration would be a necessary condition for performing patient-centered care services more frequently [22]. This underlines the need for further studies to assess the pharmacoeconomic potential of reimbursed community pharmacy services.

For polymedication, earlier pilot studies in Saxony and Thuringia have already shown positive outcomes from a structured interprofessional medication management program named ARMIN [12]. This program was performed in close cooperation with physicians in the primary care setting. In the end, mortality and hospitalization rates decreased. The ARMIN program was not continued but ultimately led to the reimbursed community pharmacy services program being examined here.

The positive benefits of this service justify the higher costs compared to services concerning inhalation and hypertension. This is particularly true because polymedication checks have been part of the hospital pharmacy services at hospital admission [23] and in discharge management [24]. But suppose the medication is subsequently modified by the general practitioner, the benefits of hospital pharmacy services may get lost and the patient is treated this way in the outpatient clinic over a longer period. In that case, it quickly becomes clear why - without calling into question the hospital pharmacy services - the involvement of the community pharmacy in the area of polymedication is indispensable. Not only drug-drug interactions but also the appropriateness of the medications for the patients are clinically relevant issues, which can be considered via the medication appropriateness index in community pharmacies [25].

In services concerning polymedication, transplantation, and anticancer drugs, pharmacists identified communication with physicians and the fear of competing with them as the major barrier. To facilitate cooperation, the wishes and needs of physicians need to be evaluated in terms of communication with pharmacies in general and concerning community pharmacy services in particular.

The barrier that patient demand is too low was also reported for services concerning transplantation and anticancer drugs. Here, the cause may differ from services concerning hypertension and inhalation. At first sight, very few patients need oral anticancer medication and immunosuppressants post-transplantation compared to those needing care for hypertension and respiratory diseases. Additionally, most patients facing cancer or organ transplantation receive treatment in specialized centers, so the consultation of a community pharmacist is much less likely. However, this assumption is contradicted by activities in the field of anti-tumor oral therapy [26] and transplantation [27] that emphasize interprofessional collaboration that includes pharmacists.

One limitation of the reimbursed services is that they are not explicitly aimed at medical and pharmaceutical collaboration, as was the case with the ARMIN project [12]. However, this is precisely what is essential for services in oral oncology, transplant medicine and the management of polymedication.

#### Solving strategies (iii)

Two main barriers reported in our survey were documentation requirements and the data protection declaration – neither can be solved by pharmacies themselves; rather, they are framework conditions addressing health care policy.

The Normalisation Process Theory (NPT) by May et al. [28] describes as one core mechanism the collective action, where the question arises which specific actions and changes taken by involved individuals can be identified in relation to the implementation of a new practice. In asking for solving strategies, it can be examined which such actions have been developed by the pharmacists. To solve the lack of time and pharmaceutical staff, about one in three pharmacists from offering pharmacies reported developing standardized procedures to perform the reimbursed community pharmacy services. Some tried to hire pharmaceutical staff but had difficulties in finding new staff. By adapting the reimbursed community pharmacy services to the needs of the community pharmacies, working in community pharmacies may become more attractive by strengthening the professional competence of pharmacists, creating more job satisfaction through specific pharmaceutical tasks, and increasing the interest of young people to study pharmacy and work in community pharmacies. In this way, the services themselves can solve several problems in the pharmacy, such as the shortage of specialists.

One in three pharmacists working in pharmacies that offer hypertension and inhalation services reported promoting these services to improve patient demand, and one in four had special communication training.

The amount of communication training for polymedication, transplantation, and anticancer drugs services is much less. For those reimbursed community pharmacy services, nearly half the pharmacists from offering pharmacies chose advanced training as a solution strategy.

In contrast, transplantation and anticancer drugs services were reported to be promoted by only one in five pharmacists from an offering pharmacy, which will likely make them fall further behind.

#### Future perspectives (iv)

Despite all these obstacles during implementation, two in three pharmacists of non-offering pharmacies can imagine implementing reimbursed community pharmacy services, especially for hypertension, inhalation and polymedication, and half the pharmacists from offering pharmacies plan to increase these services, which shows their high potential.

At least one in four pharmacists from an offering pharmacy plans to increase the amount of transplantation service and one in three plans to improve anticancer drugs service. In contrast, only 5% of the pharmacists from non-offering pharmacies plan to offer those two services. This indicates that the barriers to implementing transplantation and anticancer drugs services were considered more fundamental.

More than half of the pharmacists call for reducing bureaucracy to facilitate the implementation of reimbursed community pharmacy services, and one third of the pharmacists from an offering pharmacy wish to implement software for these services in their currently used pharmacy software.

For hypertension and inhalation services, one in three pharmacists from an offering pharmacy wishes for help in communication with patients and support in promotion, one in four wishes for support in communication with physicians, and one in four wishes for modularized on-site training.

One in four pharmacists from a non-offering pharmacy wishes for checklists on how to get started with hypertension, inhalation and polymedication services.

For polymedication, transplantation and anticancer drugs services, nearly half the pharmacists from an offering pharmacy wish for help communicating with physicians. This underlines the need to assess the physicians’ needs and wishes to develop a common approach.

### Suggestions

Former studies have shown the benefit of community pharmacy services for example in patients with asthma bronchiale and hypertension as well as the benefits of Medication reviews performed by pharmacists. This justifies the necessity to provide those services on a large scale. To increase the implementation of the community pharmacy services in German community pharmacies, some suggestions for substantial changes and for further research can be derived from the results of this study.

On the one hand the framework conditions of those services need to be reconsidered. Further studies are needed to assess if providing those community pharmacy services once a year is enough to increase patient safety or if an interval adapted to individual patient’s needs would be preferable. Additionally, studies to assess the pharmacoeconomic potential and to validate the remuneration are needed. Furthermore, it should be proved from the health care political side, how the documentation effort can be reduced to facilitate the implementation of community pharmacy services.

On the other hand the communication between pharmacists and physicians needs to be improved. Former studies have shown that the medical and pharmaceutical collaboration in terms of polymedication, anticancer drugs and immunosuppressants post-transplantation is essential to achieve the maximum benefit for patients. As those services are not explicitly aimed at those collaboration, further studies need to examine the wishes and needs of physicians in terms of communication with pharmacies concerning community pharmacy services to develop a common approach.

In addition, more specific information material for patients and more targeted information campaigns would be helpful to increase patient’s awareness of the services and thus facilitate patient communication.

### International contextualisation

In order to contextualise the results of the study, they should be compared with community pharmacy services in other countries where these services have been remunerated and provided for a longer period of time.

In Australia for example several community pharmacy services are remunerated, among others the Home Medication Review. The communication between pharmacists, general practitioner, and other health care professionals is obligatory, as the Home Medication Review needs to be requested by a physician. The service of Home Medication Review can be supplemented by two follow up visits within 9 months after the consultation, but there are several reasons why an additional review can be requested [29, 30]. In Germany, the only reason for an additional review is a significant change in patient’s medication. This obligatory request from the physician and the required communication between pharmacist and physician may facilitate medical and pharmaceutical collaboration. This is comparable to the findings in the ARMIN-Project [12]. In the German concept of remunerated community pharmacy services on the other hand this collaboration has not been made obligatory.

In Ontario, there is a start-up fee that pharmacies can claim for their first Medication review to facilitate the implementation of Medication reviews. After this they get paid per Medication review provided [31]. If such a start-up fee would make the community pharmacy services more attractive from a pharmacoeconomic point of view needs to be examined.

A review for Medication reviews in community pharmacies in Switzerland revealed, that the implementation of those services is progressing very slowly. They experienced similar problems to our survey like a lack of time and missing understanding from patients. Additionally, they experienced, that the pharmacist’s motivation rises after the first Medication review [32].

In Denmark, advices on inhalation technique can be provided every time a patient hands a prescription for an inhalation device to his community pharmacy [33, 34]. This gives the opportunity to adapt, other than in Germany, the frequency of the community pharmacy service to the patient’s needs.

The results of studies in other countries as well as their solving strategies should be kept in mind in further developing community pharmacy services in Germany.

### Limitations

When drawing conclusions from this study for an international audience, some limitations should be kept in mind: First, the real world data may differ somewhat from our results since non-offering pharmacies may be less willing to participate in a survey. By the end of 2023 6,284 German community pharmacies offered community pharmacy services [35], which is about 36%. In our survey about 81% of the participating pharmacists worked in offering pharmacies. A reason for this bias may be if pharmacies lack sufficient pharmaceutical staff and time and therefore do not offer community pharmacy services, the staff may not have the time to participate. Additionally, if pharmacies fundamentally oppose reimbursed services, they may not be willing to deal with the issue.

Secondly, it should be borne in mind that our survey can only provide a snapshot of the current situation. The results show that the processes are in flux and that many more pharmacies will likely agree to offer such services. This makes it all the more important to use the survey results to better support those pharmacies that are willing to offer such a service and to convince the others who are perhaps not yet ready to do so.

Thirdly, it should be noted that although we offered the survey to pharmacy chambers in all federal states, a few chambers decided not to participate. There may be various reasons for this, but it may also be because the implementation rates in these states may not yet have reached the level desired by the chambers.

Lastly, we asked pharmacies to ensure that only one employee from each pharmacy participated in the survey, but this cannot be conclusively proven because of the anonymity.

## Conclusion

As seen in different studies, the involvement of the pharmacist in a patient’s therapy has many benefits for the patient. Therefore, reimbursed community pharmacy services have been implemented in 2022 in German community pharmacies. As shown by the results of this study, there are many pharmacists who already offer those services, but some pharmacists have not yet offered them. This study has shown that the barriers to offer community pharmacy services highly differ depending on the specific reimbursed community pharmacy service, but some general barriers were found to the implementation of all services: bureaucracy needs to be reduced. Additionally, the patients need to be better informed about the possibility of receiving reimbursed community pharmacy services, and pharmacies need better tools to communicate those services to their patients. Another significant barrier is the collaboration of pharmacists and physicians. Therefore, additional studies are needed to understand how to improve those collaboration to achieve maximum benefits for the patients.

Several pharmacists developed supportive solutions to overcome the barriers within the implementation process on their own, such as checklists on how to get started and modularized on-site trainings and communication training. Those developments should be further supported.

Given the high patient benefit of such services described in the literature, reimbursable community pharmacy services have great potential for further development. Therefore, customized support regarding the needs of the pharmacists should be provided since most pharmacists who do not yet offer these services today can imagine offering them in the future.

The results of this study show the need for further studies concerning the potential frequency of offer, the pharmacoeconomic potential and the wishes and needs of physicians to improve the medical and pharmaceutical collaboration in terms of community pharmacy services.

## Electronic supplementary material

Below is the link to the electronic supplementary material.

Supplementary Material 1.

Supplementary Material 2.

## Data Availability

The datasets generated during and analyzed during the current study are available from the corresponding author on reasonable request.

## Declarations

The pharmacists were asked whether they would like to take part in this study. By starting the survey participants signed their informed consent to the anonymous data collection. They were only enrolled after they had given their consent to participate.
