# Supplementary material for: Implementation, barriers, solving strategies and future perspectives of reimbursed community pharmacy services - a nationwide survey for community pharmacies in Germany
Source: BMC Health Serv Res. 2024 Nov 25;24:1463. doi: 10.1186/s12913-024-11745-y (PMC11590365; doi:10.1186/s12913-024-11745-y)
Supplement: Supplementary file 1 — Supplementary Material 1. [file 12913_2024_11745_MOESM1_ESM.docx]

Community Pharmacy Services in German Community Pharmacies

Welcome to our survey concerning the implementation of reimbursed community pharmacy services in community pharmacies. We want to find out about the implementation of community pharmacy services and about barriers to implement them.

Therefore we want to ask you questions about your implementation, barriers in implementing and your wishes for support for the future. The participation in this survey is totally anonymous and takes about 15-20 minutes.

**Thank you for your participation!**

If you have any questions, we are happy to help you at any time. Please contact Ann-Christin Krönert.

Sincerely the Employees of the project.

**Head of the research project**

Prof. Dr. Thilo Bertsche,

Institut for Pharmacy, Department of Clinical Pharmacy, Leipzig University, Brüderstraße 32, 04103 Leipzig,

[thilo.bertsche@uni-leipzig.de](mailto:thilo.bertsche@uni-leipzig.de)

**Contact person**

Ann-Christin Krönert

Institut for Pharmacy, Clinical Pharmacy, Leipzig University, Brüderstraße 32, 04103 Leipzig,

[ann-christin.kroenert@uni-leipzig.de](mailto:ann-christin.kroenert@uni-leipzig.de)

Please answer this survey only one time per community pharmacy.

If you take part in a branch network, please ask the questions regarding your community pharmacy, not for the whole branch network. Every community pharmacy of the branch network should participate for themselves, even if they do not offer any reimbursed community pharmacy services by now.

You can skip single questions, if you do not want to answer them and you can cancel the participation at any time of the survey.

- I have read and agree to the privacy policy.

**Does your community pharmacy offer any reimbursed community pharmacy services?**

- Yes
- No

**Have you been offering any community pharmacy services before they got reimbursable in 2022?** *(If first question was “yes”)*

- Blood pressure measurement
- Detailed guide for inhalation techniques using demo inhalers
- Drug interaction check
- Medication analyses (inclusive brown-bag-analysis and structured patient advise
- Counseling in organ transplantation or immunosuppressive therapy
- Counseling in oral anticancer drug therapy
- Blood sugar measurement
- No
- Other:__________

**Which reimbursed community pharmacy services do you offer?** *(If first question was “yes”)*

- Blood pressure control in hypertension
- Assuring proper inhalation techniques for patients receiving a new device or a device change
- Medication review for patients with polymedication
- Medication review with follow-up for patients taking immunosuppressants post-transplantation
- Medication review with follow-up for patients taking oral anticancer drugs

**Do you want to change your offer in the future?** *(If first question was “yes”)*

|  | Hypertension | Inhalation | Polymedication | Transplantation | Anticancer drugs |
| --- | --- | --- | --- | --- | --- |
| Planning to increase |  |  |  |  |  |
| Would like to increase but no concrete planning |  |  |  |  |  |
| Planning to restrict the offer |  |  |  |  |  |
| Do not want to offer |  |  |  |  |  |
| No answer |  |  |  |  |  |
| Satisfied with the actual offer |  |  |  |  |  |

**Do you offer any similar services to the reimbursed community pharmacy services?** *(If first question was “no”)*

- Blood pressure measurement
- Detailed guide for inhalation techniques using demo inhalers
- Drug interaction check
- Medication analyses (including brown-bag-analysis and structured patient advise)
- Counseling in organ transplantation or immunosuppressive therapy
- Counseling in oral anticancer drug therapy
- Blood sugar measurement
- No
- Other:__________

**Do you want to offer any reimbursed community pharmacy services in the future?** *(If first question was “no”)*

|  | Hypertension | Inhalation | Polymedication | Transplantation | Anticancer drugs |
| --- | --- | --- | --- | --- | --- |
| Planning to increase |  |  |  |  |  |
| Would like to increase but no concrete planning |  |  |  |  |  |
| Do not want to offer |  |  |  |  |  |
| No answer |  |  |  |  |  |
| Satisfied with the actual offer |  |  |  |  |  |

**Which barriers did you face in implementing reimbursed community pharmacy services?**

|  | Hypertension | Inhalation | Polymedication | Transplantation | Anticancer drugs |
| --- | --- | --- | --- | --- | --- |
| No interest in topic |  |  |  |  |  |
| Further (voluntarily) advanced trainings needed |  |  |  |  |  |
| Lack of staff with mandatory training |  |  |  |  |  |
| Too little remuneration |  |  |  |  |  |
| Lack of time |  |  |  |  |  |
| High documentation requirements |  |  |  |  |  |
| Lack of pharmaceutical staff |  |  |  |  |  |
| Letting patients fill in the data protection declaration |  |  |  |  |  |
| Communication with patients |  |  |  |  |  |
| Too little patient demand |  |  |  |  |  |
| Communication with physicians |  |  |  |  |  |
| Fear of competing with physicians |  |  |  |  |  |
| No or to little space |  |  |  |  |  |
| Patients do not consent to the release from duty of confidentiality for consultation with the physician |  |  |  |  |  |
| Interface problems (no program for scheduling, patient data and billing) |  |  |  |  |  |
| Too little capacity for excessive demand |  |  |  |  |  |
| None |  |  |  |  |  |
| Other |  |  |  |  |  |

Other:___________________

**Which solving strategies have you developed to facilitate the implementation of the reimbursed community pharmacy services?**

|  | Hypertension | Inhalation | Polymedication | Transplantation | Anticancer drugs |
| --- | --- | --- | --- | --- | --- |
| Hiring pharmaceutical staff |  |  |  |  |  |
| Developing standardized procedure |  |  |  |  |  |
| Communication training |  |  |  |  |  |
| Advanced training |  |  |  |  |  |
| Promotion |  |  |  |  |  |
| Rent extra room |  |  |  |  |  |
| No intervention |  |  |  |  |  |
| Other |  |  |  |  |  |

Other:___________________

**Which further support would you wish for in the future to increase the number of reimbursed community pharmacy services offered?**

|  | Hypertension | Inhalation | Polymedication | Transplantation | Anticancer drugs |
| --- | --- | --- | --- | --- | --- |
| Modularized on-site training |  |  |  |  |  |
| Experience report from other community pharmacies |  |  |  |  |  |
| Exchange of experiences |  |  |  |  |  |
| Checklist how to get started |  |  |  |  |  |
| Checklist for operating |  |  |  |  |  |
| Help in communication with patients |  |  |  |  |  |
| Help in communication with physicians |  |  |  |  |  |
| Help in promotion |  |  |  |  |  |
| Less bureaucracy |  |  |  |  |  |
| Special software for reimbursed community pharmacy services |  |  |  |  |  |
| Implementing software for community pharmacy services in pharmacy software |  |  |  |  |  |
| Non |  |  |  |  |  |
| Other |  |  |  |  |  |

Other:___________________

**Which further community pharmacy services would you offer, if they became reimbursable?**

_____________________________________________________________________________

**In which chamber of pharmacists is your community pharmacy located?**

- Baden-Wuerttemberg
- Bavaria
- Berlin
- Bremen
- Brandenburg
- Hamburg
- Hesse
- Mecklenburg-Western Pomerania
- Lower Saxony
- North Rhine
- Westphalia-Lippe
- Rhineland-Palatinate
- Saarland
- Saxony
- Saxony-Anhalt
- Schleswig-Holstein
- Thuringia
- No answer

**Thank you for participating!**
