# Supplementary material for: Implementation, barriers, solving strategies and future perspectives of reimbursed community pharmacy services - a nationwide survey for community pharmacies in Germany
Source: BMC Health Serv Res. 2024 Nov 25;24:1463. doi: 10.1186/s12913-024-11745-y (PMC11590365; doi:10.1186/s12913-024-11745-y)
Supplement: Supplementary file 2 — Supplementary Material 2. [file 12913_2024_11745_MOESM2_ESM.docx]

*Supplemental Table 1: Full list of barriers in absolute numbers (multiple categories were possible)*

| Barriers | Hypertension | | Inhalation | | Polymedication | | Transplantation | | Anticancer drugs | |
| --- | --- | --- | --- | --- | --- | --- | --- | --- | --- | --- |
|  | offering | non-offering | offering | non-offering | offering | non-offering | offering | non-offering | offering | non-offering |
| No interest in topic | 3 | 1 | 3 | 1 | 1 | 1 | 1 | 21 | 2 | 19 |
| Further (voluntarily) advanced trainings needed | 21 | 6 | 26 | 11 | 23 | 15 | 2 | 34 | 5 | 33 |
| Lack of staff with mandatory training | Not relevant* | Not relevant* | Not relevant* | Not relevant* | 36 | 28 | 6 | 37 | 7 | 38 |
| Too little remuneration | 34 | 17 | 26 | 19 | 58 | 19 | 8 | 20 | 8 | 20 |
| Lack of time | 76 | 27 | 79 | 31 | 103 | 35 | 8 | 28 | 13 | 29 |
| High documentation requirements | 76 | 35 | 75 | 33 | 83 | 34 | 9 | 37 | 13 | 36 |
| Lack of pharmaceutical staff | 68 | 23 | 68 | 25 | 83 | 29 | 6 | 29 | 9 | 31 |
| Letting patients fill in the data protection declaration | 83 | 24 | 83 | 24 | 55 | 23 | 10 | 21 | 10 | 23 |
| Communication with patients | 53 | 7 | 54 | 9 | 45 | 9 | 5 | 10 | 7 | 9 |
| Too little patient demand | 66 | 8 | 61 | 9 | 38 | 15 | 16 | 46 | 18 | 42 |
| Communication with physicians | 33 | 9 | 35 | 6 | 80 | 21 | 8 | 17 | 13 | 15 |
| Fear of competing with physicians | 24 | 15 | 24 | 11 | 67 | 19 | 6 | 24 | 11 | 20 |
| No or to little space | 3 | 1 | 4 | 2 | 6 | 3 | 0 | 4 | 1 | 3 |
| Patients do not consent to the release from duty of confidentiality for consultation with the physician | 9 | 5 | 9 | 3 | 28 | 7 |  | 6 | 2 | 7 |
| Interface problems (no program for scheduling, patient data and billing) | 17 | 4 | 16 | 6 | 18 | 8 | 4 | 10 | 5 | 9 |
| Too little capacity for excessive demand | 20 | 6 | 19 | 8 | 40 | 10 | 3 | 15 | 6 | 13 |
| None | 4 | 0 | 3 | 0 | 2 | 0 | 1 | 1 | 1 | 1 |
| Other | 2 | 4 | 1 | 4 | 2 | 4 | 0 | 5 | 0 | 5 |

* The point “Lack of staff with mandatory training” is not relevant for hypertension and inhalation as there are no mandatory trainings needed to offer those two services.

*Supplemental Table 2: Solving strategies in absolute numbers (multiple categories were possible)*

| Solving strategies | Hypertension | | Inhalation | | Polymedication | | Transplantation | | Anticancer drugs | |
| --- | --- | --- | --- | --- | --- | --- | --- | --- | --- | --- |
|  | offering | non-offering | offering | non-offering | offering | non-offering | offering | non-offering | offering | non-offering |
| Hiring pharmaceutical staff | 5 | 2 | 4 | 2 | 9 | 1 | 0 | 0 | 1 | 0 |
| Developing standardized procedure | 54 | 4 | 57 | 3 | 52 | 4 | 7 | 6 | 12 | 4 |
| Communication training | 38 | 4 | 40 | 3 | 24 | 3 | 1 | 2 | 1 | 4 |
| Advanced training | 34 | 5 | 51 | 5 | 85 | 8 | 10 | 8 | 13 | 8 |
| Promotion | 49 | 2 | 50 | 2 | 55 | 3 | 5 | 2 | 7 | 2 |
| Rent extra room | 0 | 0 | 0 | 0 | 2 | 0 | 0 | 0 | 0 | 0 |
| No intervention | 28 | 23 | 20 | 26 | 16 | 27 | 2 | 40 | 3 | 39 |
| Other | 4 | 2 | 4 | 2 | 3 | 2 | 0 | 2 | 0 | 2 |

*Supplemental Table 3: Wishes for support in absolute numbers (multiple categories were possible)*

| Wishes | Hypertension | | Inhalation | | Polymedication | | Transplantation | | Anticancer drugs | |
| --- | --- | --- | --- | --- | --- | --- | --- | --- | --- | --- |
|  | offering | non-offering | offering | non-offering | offering | non-offering | offering | non-offering | offering | non-offering |
| Modularized on-site training | 42 | 8 | 44 | 12 | 28 | 14 | 2 | 16 | 3 | 16 |
| Experience report from other community pharmacies | 22 | 9 | 23 | 10 | 33 | 11 | 6 | 17 | 7 | 18 |
| Exchange of experiences | 23 | 6 | 24 | 8 | 45 | 10 | 5 | 16 | 5 | 18 |
| Checklist how to get started | 31 | 12 | 31 | 14 | 34 | 16 | 7 | 16 | 8 | 16 |
| Checklist for operating | 29 | 10 | 32 | 11 | 34 | 12 | 8 | 10 | 11 | 9 |
| Help in communication with patients | 53 | 10 | 50 | 12 | 56 | 9 | 6 | 16 | 8 | 16 |
| Help in communication with physicians | 40 | 13 | 44 | 13 | 68 | 16 | 11 | 19 | 14 | 18 |
| Help in promotion | 50 | 3 | 51 | 4 | 51 | 6 | 6 | 12 | 9 | 9 |
| Less bureaucracy | 105 | 34 | 107 | 34 | 102 | 34 | 14 | 50 | 18 | 50 |
| Special software for reimbursed community pharmacy services with schedule | 28 | 9 | 26 | 11 | 35 | 10 | 4 | 14 | 7 | 12 |
| Implementing software for community pharmacy services in pharmacy software | 50 | 13 | 48 | 16 | 58 | 10 | 7 | 19 | 11 | 17 |
| Non | 9 | 2 | 6 | 4 | 7 | 2 | 1 | 11 | 0 | 11 |
| Other | 1 | 6 | 1 | 6 | 2 | 6 | 1 | 6 | 1 | 6 |
